# *In situ* growth of carbon nanotubes on MXenes for high-performance electromagnetic wave absorption†

**DOI:** 10.1039/d5ra03991f

**Published:** 2025-07-24

**Authors:** Zhichao Mu, Lanzhi Wang, Benhui Fan, Zuojuan Du, Jianling Yue, Yu Liu, Xiaozhong Huang

**Affiliations:** a State Key Laboratory of Powder Metallurgy, Powder Metallurgy Research Institute, Central South University Changsha 410083 PR China yu_liu@csu.edu.cn; b Hunan Key Laboratory of Advanced Fibers and Composites, Central South University Changsha Hunan 410083 PR China; c Beijing Institute of Aerospace Launch Technology Beijing 100076 PR China; d Cerema, Research Team 10 chemin de la Poudrière La Grand-Quevilly 76120 France

## Abstract

Two-dimensional transition metal carbides and nitrides (MXenes), especially titanium carbide, are ideal materials for high-performance microwave absorbers. Nonetheless, their characteristics of being prone to stacking and agglomeration seriously affect their application. Moreover, their elevated electrical conductivity results in the reflection of electromagnetic waves (EMW) rather than their absorption. This study proposes a simple strategy to grow CNTs on the surface of MXenes by chemical vapor deposition (CVD) technology, and introduces a SiO_2_ intermediate layer to uniformly distribute CNTs on the substrate surface. Controlling the growth of CNTs by adjusting the reaction time to regulate the microstructure and electromagnetic parameters of the composite materials, the wave absorption performance under low filling amount was significantly improved. MXene@SiO_2_–CNTs exhibit a minimum reflection loss of −48.38 dB at a thickness of 2.1 mm, with an effective absorption bandwidth extending to 5.47 GHz (from 12.53 GHz to 18 GHz). The radar cross-section values of MXene@SiO_2_–15CNTs are all below −15 dBm^2^, significantly diminishing the likelihood of radar detection of the target.

## Abstract 2

The MXene@SiO_2_–CNTs composite, with uniform carbon nanotube coating, achieves an exceptional minimum reflection loss (−48.38 dB) and broad absorption bandwidth (5.47 GHz) at only 2.1 mm thickness, while its radar cross-section values below −15 dBm^2^.

## Graphical abstract

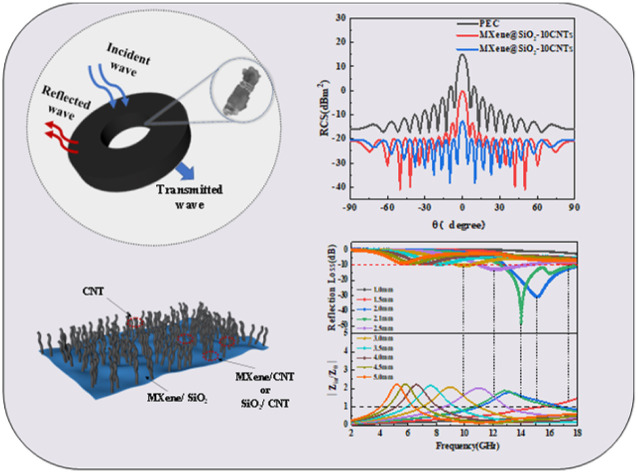

## Introduction

The swift progress of electronic information technology has led to electromagnetic pollution becoming a serious concern, driving the urgent need for advanced electromagnetic shielding and absorption materials in both civilian and military applications.^1,2^ Various types of materials have been developed, including carbon-based nanomaterials, metallic oxide nanoparticles, *etc.* Among them, the Ti_3_C_2_T_*x*_, an emerging 2D material, with excellent physical and chemical properties, shows a great prospect of application in the field of electromagnetic protection.^3–7^ The MXene films with high crystallinity has an electrical conductivity as high as 18 000 S cm^−1^, enabling the material to effectively attenuate electromagnetic waves through reflection mechanisms and achieve efficient electromagnetic shielding.^8,9^ In addition, the abundant functional groups (such as –OH, –O, –F) on the surface of MXene make it easy to undergo surface modification, providing multiple approaches for regulating electromagnetic properties and solving the common impedance mismatch problem in highly conductive materials.^10–13^

However, the low dielectric loss and the easy stacking between the layers of MXene are not favorable for secondary dissipation of induced electromagnetic waves and impedance matching, which constrains its application in the microwave absorption area.^14^ At a filler content of 50%, the RL_min_ value of the Ti_3_C_2_T_*x*_ nanosheet-filled composite is only −17 dB.^15^ A strategy has been mooted to enhance the microwave absorption properties of Ti_3_C_2_T_*x*_ by varying its microscopic morphology. Fan *et al.*^16^ increased the etching time of MXene nanosheets thereby increasing the interlamellar space, the maximum reflection loss reached −36.3 dB as prolonging the etching time to 3 h. Xu *et al.*^17^ modified the surface of Ti_3_C_2_T_*x*_ with different functional groups to obtain ultra-thin nanosheets, which achieved an effective absorption bandwidth (EAB) of 4.4 GHz with the thin thickness of 1.26 mm.^18^

By modifying the surface of Ti_3_C_2_T_*x*_, it can effectively resist the interlamellar stacking. Li *et al.*^19^ found that the RL_min_ value reaches −56.76 dB and the EAB is 1.87 GHz when the MXene filler is 50% and the thickness is 3.5 mm through the particle swarm optimisation (PSO) algorithm for the optimisation calculation and analysis of single multilayer Ti_3_C_2_T_*x*_ particles. However, a high absorbent concentration in the composites is usually necessary to achieve sufficient dielectric loss, which limits its application. Combining Ti_3_C_2_T_*x*_ with other nanomaterials is an effective method to reduce its concentration in composites. Cui *et al.*^20^ physically assembled carboxylated carbon nanotubes (C-CNTs) with Ti_3_C_2_ MXene nanosheets using ultrasonic spraying technique to obtain porous microspheres with minimum reflection loss of −45 dB when the optimum mass ratio was MXene : CNTs = 3 : 1 and filler content was 30%. Tong *et al.*^21^ successfully synthesised a multilayer sandwich heterostructure Ti_3_C_2_T_*x*_ MXene/PPY modified with polypyrrole chains by *in situ* chemical oxidative polymerisation, the composite consisting of 25 wt% titanium carbide/PPy hybrids in paraffinic matrix showed an RL_min_ value of −49.2 dB and a EAB_max_ value of 4.9 GHz at a thickness of 3.2 mm. Likewise, Li *et al.*^22^ fabricated lightweight MXene/PI aerogel nanocomposites (MPs) without magnetic properties by freeze-drying, in which the ‘PI chains’ anchored the MXene layer to enhance the interactions between the titanium carbide nanosheets while effectively reducing the self-stacking of the MXene layer, which exhibited excellent microwave absorption properties. The EAB attains 6.9 GHz when the MP content is 30% and the thickness is 2.5 mm.

In addition, the construction of three-dimensional conductive networks through the nanomaterial structural design is also an efficient measure to improve the impedance matching to enhance the microwave absorption performance.^23,24^ Carbon nanotubes (CNTs), as a low-dimensional carbon material with tunable surface properties, light weight, high carrier mobility (∼100 000 cm^2^ V^−1^ s^−1^) and high thermal conductivity (∼3000 W m^−1^ K^−1^), is widely used as loading materials in the MA field.^25–27^ Zhou *et al.*^28^ deposited CoNi/N–CNTs on the surface of 2D MXene lamellae by electrostatic self-assembly to obtain 3D superstructured nanocomposites. Benefitting from the synergistic effect of the 3D conductive network formed by 1D N–CNTs and the multiple loss mechanisms, the material shows an RL_min_ of −52.64 dB at the thickness of 3.8 mm, when the EAB value is 0.72 GHz. Similarly, Zou *et al.*^29^ prepared Co@C/MXene with a three-dimensional structure by growing Co@CNTs *in situ* on MXene and optimised the impedance matching performance by varying the amount of MXene. The RL_min_ of the absorber reached −50.5 dB when the MXene content in the absorber was 10 mg and the filler content was 20%, and the matching thickness was 4.0 mm. To fulfil the high requirements of lightness, broadband and high-efficiency, Li *et al.*^30^ developed a more intricate three-dimensional structure by combining CNTs, MXene, and graphene oxide (GO). The resulting absorber demonstrated an impressive RL_min_ value of −57.6 dB with an EAB of 3.3 GHz at a filler loading of 25 wt%. In our previous work, the chemical vapor deposition (CVD) was employed to grow CNTs on the surface of particles, fibers, and carbon foam, *etc.*, to form various types of hybrids.^31–34^ By adjusting the reaction conditions, the growth density and length of CNTs can be well controlled. The hybrids can form a conductive network by CNTs overlapping with each other at a low concentration in the composite, which effectively reduced the absorbent concentration.

Based on this idea, we propose the fabrication of MXene/SiO_2_–CNTs hybrids with 3D hierarchical structures by CVD. Silica (SiO_2_) is employed due to its semiconductive properties, which help reduce the conductivity of the composites and enhance their dielectric loss by introducing more interfaces. Notably, CVD enables precise control over composite structure through parameter optimization, ensuring uniform carbon nanotube distribution and strong covalent bonding of MXene/CNT, which cannot be achieved through non-*in situ* mixing.^35^ The 3D structure of the synthesized MXene/SiO_2_–CNTs hybrids introduces various multiscale interfaces. These interfaces play a crucial role in trapping and attenuating EMW energy when EMW penetrates the absorber. With a low filler content of 15% and the thickness of 2.1 mm, the absorber reaches an RL_min_ of −48.38 dB and an impressive EAB of 5.47 GHz, highlighting its unique potential for lightweight and broadband EMW absorbers.

## Experimental section

### Materials

MXene (Ti_3_C_2_T_*x*_) was purchased from XinXi Technology. Tetraethyl orthosilicate (TEOS), ferrocene, xylene and other reagents were procured from Sinopharm Chemical Reagent Co., Ltd, and all chemical reagents used were of analytical purification. The gas required for chemical vapor deposition was high-purity gas (99.99% by volume), purchased from Changsha Gaoke Gas Co., Ltd.

### Samples preparation

**Fig. 1:**
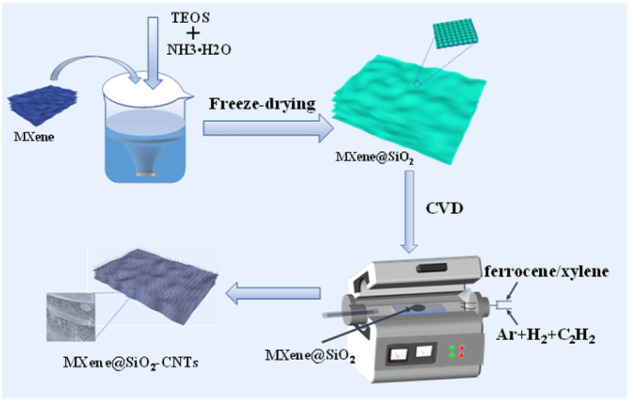
The schematic diagram of the synthesis process of MXene@SiO_2_–CNTs composites.

The fabrication process of MXene@SiO_2_–CNTs composites was shown in Fig. 1. First, 0.5 g of MXene was added to a mixture of 400 mL anhydrous ethanol and 50 mL deionized water and dispersed by sonication for 30 minutes. Ammonia solution was then slowly added to the MXene dispersion under mechanical agitation to adjust the pH to 8. Subsequently, 1.5 mL of tetraethyl orthosilicate (TEOS) was introduced to the mixture, which was then stirred at room temperature for 20 hours. Ultimately, the samples were extracted from the solution, rinsed twice with ethanol, and once with deionized water. The cleaned precipitate was then re-dispersed in 10 mL of deionized water using ultrasonic treatment for 5 minutes. The dispersion was then transferred to a beaker and freeze-dried for 48 hours to obtain MXene@SiO_2_.

The hybrids were fabricated by CVD. The MXene@SiO_2_ was placed in a tube furnace, and the heating process began by passing a mixture of argon (Ar) and hydrogen (H_2_) gases at a volume ratio of 4 : 1. Once the furnace temperature reached 750 °C and stabilized for 5 minutes, a 0.05 mg per mL ferrocene/xylene solution was introduced as the catalyst, and 20 sccm of acetylene (C_2_H_2_) was fed into the chamber as the carbon source. The growth of CNTs was controlled by varying the reaction time to 5, 10, and 15 minutes, respectively. The SiO_2_-coated MXene–CNTs hybrid materials (MXene@SiO_2_–CNTs) were then obtained. The samples were named MXene@SiO_2_–5CNTs, MXene@SiO_2_–10CNTs, and MXene@SiO_2_–15CNTs, based on the growth time of CNTs. For comparison, CNTs were grown on the surface of MXene without SiO_2_ coating (reaction time: 15 minutes) by the same process conditions. This sample was designated MXene–15CNTs.

### Characterization

The elemental composition of the samples were characterized using X-ray diffraction (XRD, Rigaku SmartLab SE, Japan). To examine the micromorphology and surface elemental distribution, field emission scanning electron microscopy (SEM, TESCAN MIRA3 LMU) integrated with an energy dispersive spectrometer (EDS) was utilized. Additionally, thermogravimetric analysis (TGA, TA TGA 550, USA) was conducted to quantify the elemental content in the samples. The lattice defect and graphitization degree of the samples were characterized by a Raman spectrometer with a 532 nm excitation laser. The electromagnetic parameters were evaluated by a vector network analyzer (VNA, Agilent 8720ET) over the frequency range of 2–18 GHz. For electromagnetic measurements, the composite material was mixed with paraffin at a filler loading of 15 wt% and compressed into a coaxial ring with an inner diameter of 3.04 mm and an outer diameter of 7 mm.

## Results and discussion

### The microstructure of MXene@SiO_2_ and MXene@SiO_2_–CNTs hybrids

**Fig. 2:**
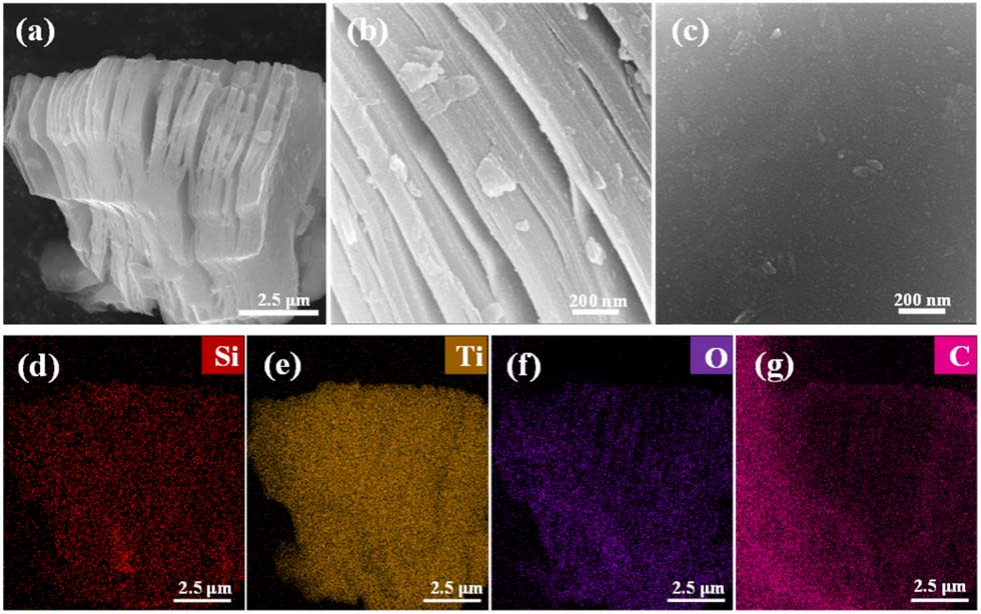
SEM images of MXene@SiO_2_ (a–c), EDS of images of Si, Ti, O, C elements for MXene@SiO_2_ composites: (d–g).

As shown in Fig. S1,† CNTs do not grow well on pure MXene. To ensure the uniform growth of CNTs on the MXene surface, SiO_2_ was used as an intermediate layer. Fig. 2 shows the microscopic morphology and EDS results of MXene@SiO_2_. In Fig. 2(a)–(c), the presence of SiO_2_ uniformly distributed on the surface of MXene. Additionally, compared to pure MXene (Fig. S2†), the shape of MXene@SiO_2_ did not change significantly, which can be attributed to the low TEOS addition and the low alkalinity of the solvent during the SiO_2_ deposition process. To further characterize the distribution of SiO_2_, EDS as employed to characterize the elemental composition of the MXene@SiO_2_ and pure MXene. The results are presented in Fig. 2(d)–(g) and S2(c)–(f)† and summarized in Table S1.† Pure MXene is mainly composed of three elements, Ti (11%), C (70.2%), and O (18.7%). While Fig. 2(d) shows that the Si element content reaches 0.5%, this result also indicates that the silica particles are successfully attached to the surface of the MXene matrix. The additional C and O elements listed in Table S1† are attributed to the sample's exposure to air and the use of conductive adhesive during sample preparation and transportation for testing.

**Fig. 3:**
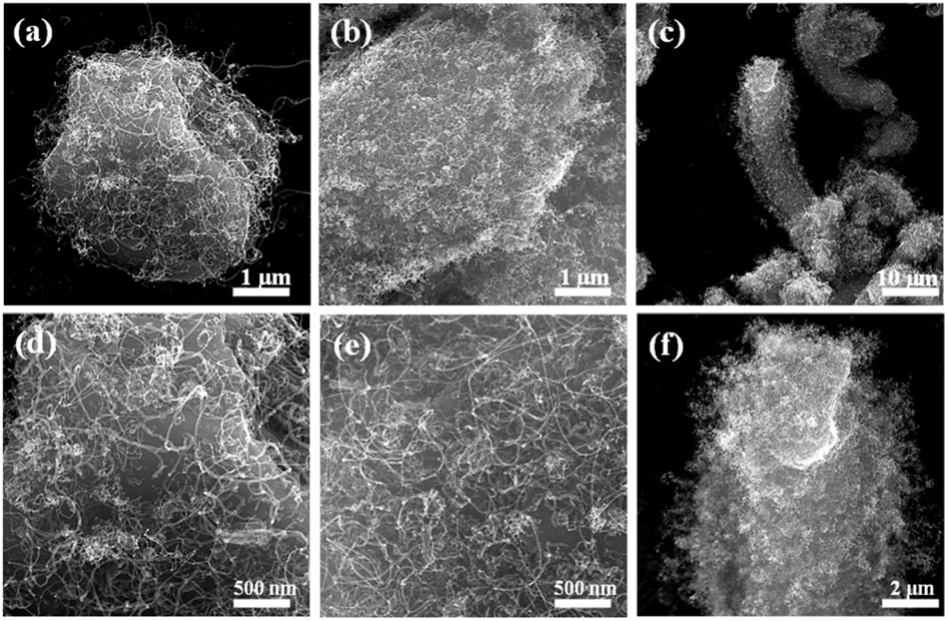
SEM images of MXene@SiO_2_–CNTs composites with reaction time of 5 min (a and d), 10 min (b and e), 15 min (c and f).

The microscopic morphology of the hybrids which were prepared under different reaction conditions was examined by SEM, as shown in Fig. 3. As shown in Fig. S1(a) and (b),† the growth of CNTs on pure MXene was less and unevenly distributed. The growth of carbon nanotubes was significantly improved after the addition of silica intermediate layer. The uniformly coated hybridized particles of carbon nanotubes were obtained when the reaction time was 5 min with an average tube diameter of approximately 18 nm.

Notably, CNTs grow not only on the surface of the MXene@SiO_2_ composite but also within its interlayers. Fig. 3 illustrates that the concentration of carbon nanotubes escalates with prolonged reaction time. The proliferation of CNTs undermines the interlayer forces of MXene, leading to alterations in the lamellar spacing of MXene and its subsequent reconstruction based on its original morphology. At the reaction time of 10 min, carbon nanotubes proliferate extensively on the surface and interstitially within the layers of MXene, resulting in a three-dimensional spatial configuration. With the reaction time extended to 15 min, the length of hybrid particles rose markedly, exhibiting a jellyfish-like morphology with an average length of 35 μm.

The contrasting morphologies of CNTs growth on MXene substrates with and without SiO_2_ coating suggest that the presence of SiO_2_ promotes CNTs growth. This phenomenon can be attributed to the complex movement of Fe particles into the accordion-like MXene@SiO_2_ interlayers, driven by the hot gas flow during the reaction process: when ferrocene enters the tube furnace, it decomposes at high temperatures to generate catalytically active Fe particles. Due to its excellent chemical stability under high-temperature conditions, the SiO_2_ coating does not hinder the catalytic activity of Fe. Instead, it provides a stable surface that promotes the rapid initiation and uniform growth of CNTs on the substrate.^36^ Additionally, the SiO_2_ coating creates favorable conditions for the dissolution and diffusion of carbon atoms, further enhancing CNTs growth.^37^ The simultaneous formation of SiO_2_ at ambient temperatures may contribute to defect alignment, improved trapping of hydrocarbon radicals, their subsequent decomposition, and enhanced carbon solubility and diffusion. The presence of the oxide shell layer significantly increases the material's capacity to adsorb CH_*x*_ radicals and Fe particles. This, in turn, strengthens the interaction between the surface and the catalyst particles, ultimately promoting the robust growth of CNTs.^38–40^ SiO_2_ can also combine with the F-terminated groups on the surface of MXene. This combination mode is conducive to enhancing the contact of nano-hybrids, effectively preventing the coverage of conductive components, and promoting stable conductive network formation.^41^

**Fig. 4:**
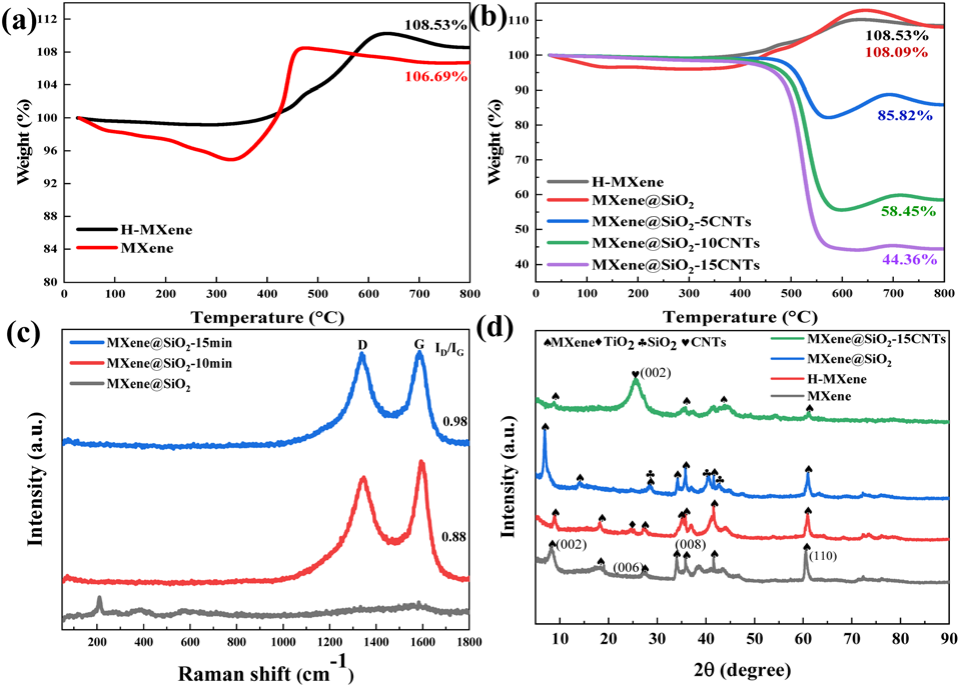
TG of MXene, H-MXene, MXene@SiO_2_ and MXene@SiO_2_–CNTs composites (a and b), Raman of MXene@SiO_2_ and MXene@SiO_2_–CNTs composites (c), XRD of MXene, H-MXene, MXene@SiO_2_ and MXene@SiO_2_–15CNTs (d).

The TG curves of MXene, H-MXene, and MXene@SiO_2_–CNTs composites synthesized at reaction times of 5, 10, and 15 minutes are presented in Fig. 4(a) and (b). The H-MXene sample (Fig. S3†) was obtained by holding the temperature at 750 °C for 5 minutes after reaching the reaction temperature. As shown in Fig. 4(a), the TG curves of H-MXene exhibit no mass loss below 400 °C, unlike untreated MXene. This stability is attributed to the heat treatment, which removes physically adsorbed water from the MXene surface as well as surface functional groups, consisting of hydroxyl (OH), oxygen (O), and a small amount of residual fluorine (F) groups from the HF etching process.^42,43^ The weight loss observed in H-MXene around 700 °C is due to the irreversible disproportionation of a small fraction of MXene, leading to the formation of cubic TiC, TiO_2_, and CO/CO_2_ gas.^44^ Additionally, the weight increases around 550 °C is due to the oxidation of the MXene surface, which generates TiO_2_. The TG curve of MXene@SiO_2_–CNTs composites (Fig. 4(b)) follows a similar trend to that of H-MXene and serves as a reference to estimate the mass fraction of CNTs in the hybrid composites. Through calculation, the mass fractions of CNTs in the composites synthesized with reaction times of 5, 10, and 15 minutes were determined to be 20.60%, 45.92%, and 58.96%, respectively. The Raman results are shown in Fig. 4(c). Compared to MXene@ SiO_2_, two typical peaks of MXene@SiO_2_–CNTs are present. The D-band near 1350 cm^−1^ and the G-band near 1580 cm^−1^ represent lattice defects in the carbon atom and telescopic vibrations in the sp^2^ hybridization plane of the carbon atom, respectively. The *I*_D_/*I*_G_ ratios represented the degree of graphitization and disorder of the carbon-based material.^45^ The *I*_D_/*I*_G_ values of the hybridized materials increased with the reaction time. This is due to the increase in the content of carbon nanotubes, which leads to changes in the local electronic structure and increases the intensity of the D peak, as well as the number of interfaces between the hybridized material and the substrate.

The XRD results are presented in Fig. 4(d). The (002) peak of MXene@SiO_2_ shifts from 9.63° to 6.92°, compared to pristine MXene, indicating an increase in layer spacing. Additionally, the presence of a characteristic SiO_2_ peak at 28.83° confirms the successful deposition of SiO_2_ onto the MXene surface. Since MXene contains a limited number of oxygen-containing functional groups, these provide only a small amount of oxygen during heat treatment, leading to the formation of a few TiO_2_ particles.^46^ Consequently, weak TiO_2_ peaks, such as the one at 25.16°, appear in the H-MXene pattern. Despite the undesired TiO_2_ phase formation, the resulting heterogeneous interfaces contribute to additional interfacial polarization losses.^47^ Moreover, characteristic peaks corresponding to MXene are still visible in the H-MXene patterns, which were sintered under a mixed nitrogen and hydrogen atmosphere. This confirms that the MXene structure remains stable under high-temperature conditions. In the XRD pattern of MXene@SiO_2_–15CNTs composites, the broad peak near 26° corresponds to the (002) crystalline facet of graphitic carbon, indicating a high degree of graphitization following the epitaxial growth of CNTs. The weakening of MXene peak intensities is attributed to reflections from the (002) crystal plane of the CNTs, signifying successful CNTs integration.^36^

### EMW absorption performance of MXene@SiO_2_–CNTs hybrids

To investigate the EMW absorption properties of MXene@SiO_2_–CNTs hybrid materials, the relative complex permittivity and complex permeability were measured across the frequency range of 2–18 GHz by the coaxial ring method at the loading concentration of 15%. MXene@SiO_2_–CNTs hybrids are nonmagnetic, resulting in lower magnetic losses compared to composites incorporating magnetic particles.^22^ Consequently, the EMW absorption mechanism in these hybrids is primarily governed by dielectric loss.

**Fig. 5:**
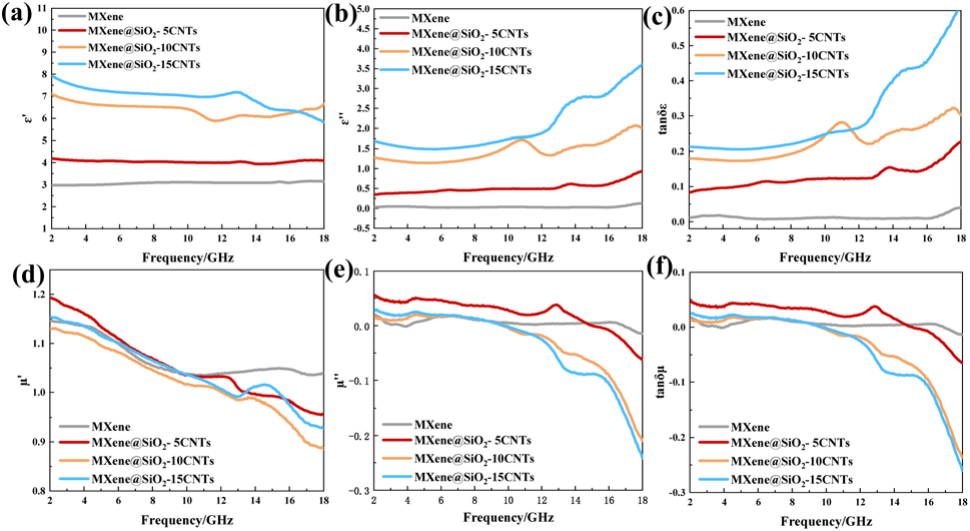
(a) Real part (*ε*′), (b) imaginary part (*ε*′′), (c) dielectric loss tangent (tan *δ*_*ε*_) for the relative complex permittivity and (d) Real part (*μ*′), (e) imaginary part (*μ*′′), (f) magnetic loss tangent (tan *δ*_*μ*_) for the relative complex permeability.

As shown in Fig. 5(a) and (b), the real part of the dielectric constant (*ε*′) for MXene remains around 3, while the imaginary part (*ε*′′) is close to 0. However, for MXene@SiO_2_–CNTs composites, *ε*′ gradually decreases with increasing frequency due to dielectric relaxation. Additionally, as the reaction time increases, the mass fraction of CNTs also increases, leading to a rise in both *ε*′ and *ε*′′. According to free electron theory, larger conductivity results in a higher *ε*′′. The incorporation of CNTs enhances the material's conductivity, thereby amplifying the conductive loss within the hybridized material.^48^ The observed increase in dielectric properties for MXene@SiO_2_–CNTs composites suggests that the application of 3D structures effectively enhances polarizations within the tested frequency range. From the view of dielectric loss generated by dipoles and interfaces, the multi-scaled interfaces formed among SiO_2_, CNTs, and MXene can trap EMW within the 3D structure, dissipating their energy through dipole resonance. This is evidenced by resonance peaks observed in the mid to high-frequency range in Fig. 5(b), likely arising from Maxwell–Wagner and dipole polarizations. From the view of conductivity loss, the incorporation of CNTs improves the material's conductive network, further increasing *ε*′′ as described by free electron theory. Moreover, the increased CNTs content alters the microscopic morphology of MXene, as seen in SEM images. The MXene lamellae interact with CNTs to form a denser, more robust 3D cross-linked skeleton, creating a superior conductive network. This structure effectively mitigates currents induced by the time-varying electromagnetic field, further enhancing the material's EMW absorption performance.^22^

Fig. 5(d) and (e) display the measured real part permeability (*μ*′) and imaginary part permeability (*μ*′′) of the samples within the frequency range of 2–18 GHz. As previously mentioned, the materials used are nonmagnetic, resulting in relatively stable magnetic properties across the tested frequency range. However, at frequencies exceeding 12 GHz, negative values for *μ*′ and *μ*′′ are observed. This phenomenon likely arises from loop currents generated within the 3D cross-linked skeleton, which induce a magnetic response opposite to the external magnetic field. When sufficient loop currents are formed, they counteract part of the external magnetic field, leading to the occurrence of negative permeability.^29^

The dielectric loss tangent (tan *δ*_*ε*_ = *ε*′′/*ε*′) and magnetic loss tangent (tan *δ*_*μ*_ = *μ*′′/*μ*′) are key parameters that reflect the EMW absorption properties of materials. As shown in Fig. 5(c), the value of tan *δ*_*ε*_ rises with increasing mass fraction of CNTs, indicating that CNTs significantly enhance the dielectric loss of the absorber. By comparing tan *δ*_*ε*_ and tan *δ*_*μ*_ (Fig. 5(f)), it is evident that MXene@SiO_2_–CNTs hybrids exhibit high dielectric loss, consistent with other MXene-based absorbers reported in the literature.^49–52^ This demonstrates the material's strong capability for dissipating EMW energy through dielectric loss mechanisms.

To evaluate the microwave absorption properties of MXene@SiO_2_–CNTs composites synthesized at different reaction times, the reflection loss values were calculated using transmission line theory. The calculation is based on the following formula:1
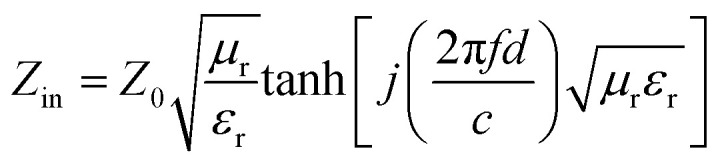
2
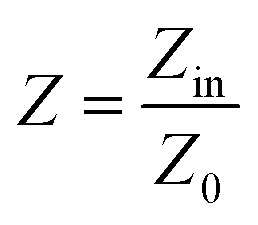
3
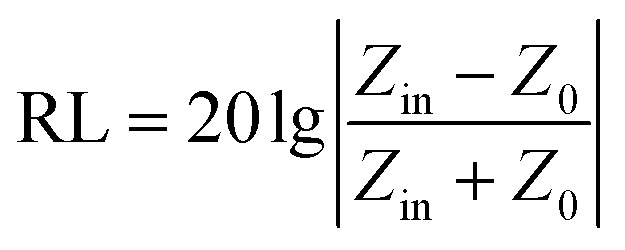
where *Z*_in_ and *Z*_0_ denote the input impedance and air impedance, respectively, and *f*, *d*, and *c* denote the frequency, thickness, and speed of light, respectively. *j* is the imaginary unit. It is commonly stated that an RL ≤ −10 dB is considered indicative of effective wave absorption, with the corresponding frequency range referred to as the EAB.

**Fig. 6:**
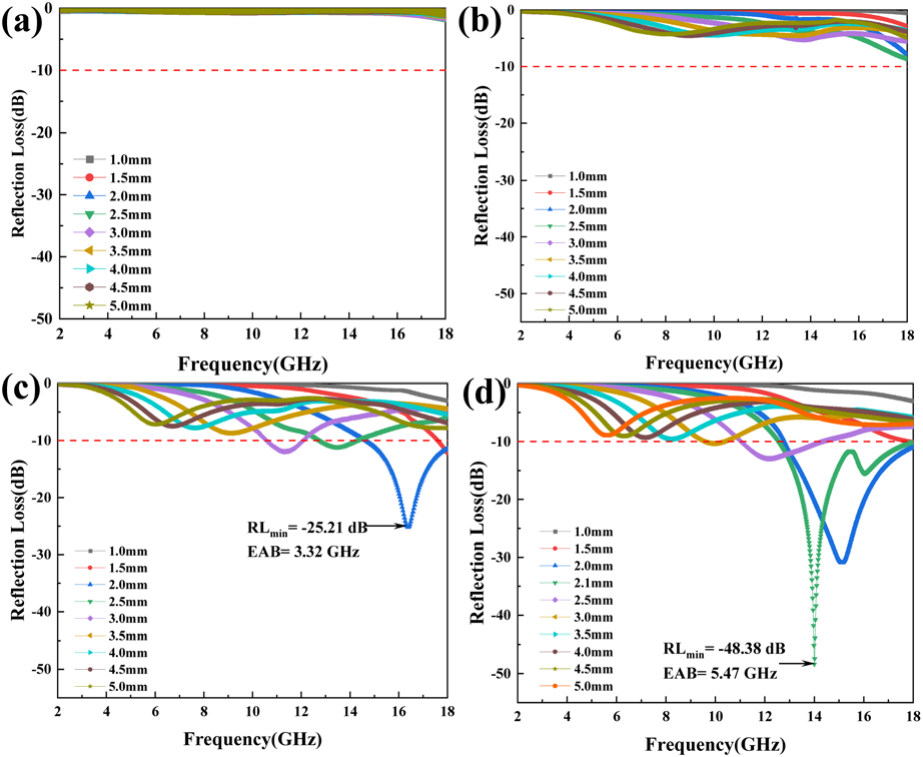
Reflection loss plots of (a) pure MXene and (b–d) MXene@SiO_2_–CNTs with reaction time of respectively 5, 10, 15 min.

Fig. 6(a)–(d) illustrate the RL curves of pure MXene, MXene@SiO_2_–5CNTs, MXene@SiO_2_–10CNTs, and MXene@SiO_2_–15CNTs at different thicknesses over the frequency range of 2–18 GHz. Pure MXene and MXene@SiO_2_–5CNTs exhibit relatively poor absorption performance, primarily due to their higher conductivity, which leads to increased reflection of EMW. In contrast, as the reaction time increases, MXene@SiO_2_–15CNTs demonstrate the best EMW absorption performance. At a low thickness of 2.1 mm, RL_min_ reaches an impressive −48.38 dB, with the maximum EAB of 5.47 GHz.

The superior microwave absorption capability of MXene@SiO_2_–CNTs hybrids is attributed to the enhanced conductive network formed by the increased CNTs content. However, as shown in Fig. 6(d) and S4,† increasing the mass loading of the absorber does not significantly improve EMW absorption performance when other process parameters remain unchanged. By the comparison of reflection loss curves of different composites, it is evident that both RL_min_ and EAB values improve significantly with longer reaction times. This improvement can be attributed to the increased CNTs content, which enhances impedance matching.^53^ However, it is important to note that higher CNTs content does not always equate to better performance. Excessive CNTs content can lead to issues such as agglomeration, which degrades the EMW absorption properties of the composite.

Additionally, an overly high CNTs content can increase conductivity, resulting in greater reflection of electromagnetic waves. This negatively impacts impedance matching, which is crucial for effective absorption. This is supported by the observed decrease in RL_min_ and EAB values in Fig. S4† when CNTs content becomes excessively high. In order to enhance the comprehensiveness of the data, the dielectric constant and performance data of MXene mechanically mixed with CNTs were tested. As shown in Fig. S5,† when the CNTs content is 50%, the dielectric constant of the sample is significantly higher than that of the MXene@SiO_2_–CNTs hybridized material, and the RL value is concentrated above −10 dB, which indicates an unsatisfactory electromagnetic wave absorption performance under the same conditions. The results indicate that *in situ* growth of carbon nanotubes on MXene matrix can effectively regulate its electromagnetic parameters and improve its conductivity loss, leading to better impedance matching and attenuation characteristics.4
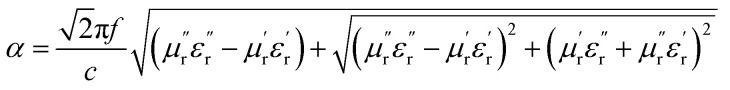


**Fig. 7:**
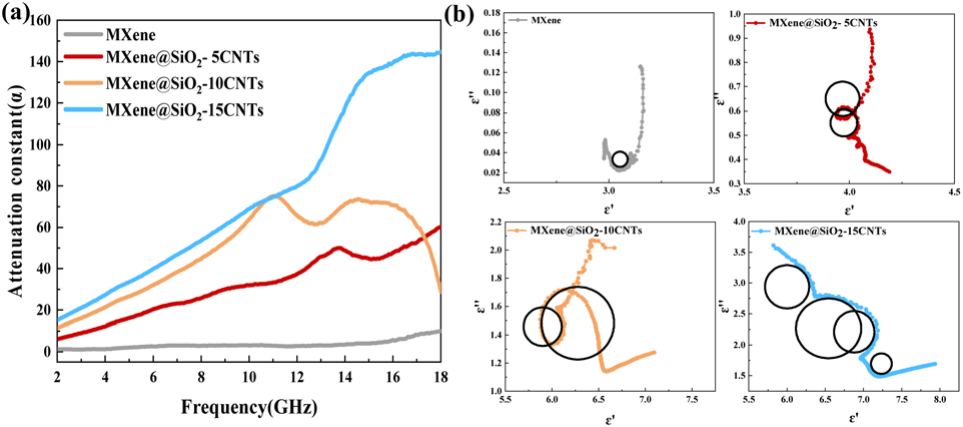
Attenuation constant (a) and Cole–Cole semicircles (b) of pure MXene and MXene@SiO_2_–CNTs composites with different growth times.

To further investigate the absorption mechanism in MXene@SiO_2_–CNTs hybrids, the *ε*′′–*ε*′ relationship curves of MXene@SiO_2_–CNTs composites in the 2–18 GHz frequency range are presented in Fig. 7(b). The Debye relaxation theory explains the synergistic effects of dipole polarization and conduction loss on the electromagnetic absorption properties of materials. Each semicircle on the *ε*′′–*ε*′ curve corresponds to a polarization process, while the linear portion represents conduction loss.^54^ For pure MXene, the Cole–Cole curve forms a single semicircle, indicating that its dielectric loss is mainly dominated by dipole polarization arising from the abundant functional groups on its surface. In contrast, MXene@SiO_2_–15CNTs exhibit multiple semicircles on their curve, signifying enhanced polarization processes. Additionally, the trailing of the curve indicates excellent conduction loss, further contributing to its superior EMW absorption performance.

**Fig. 8:**
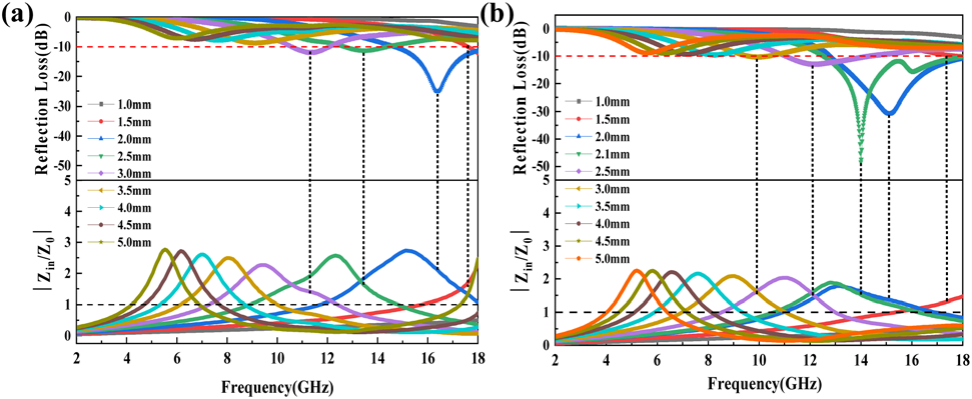
Normalized characteristic impedance (*Z*_in_/*Z*_0_) plots of MXene@SiO_2_–CNTs with growth time of respectively 10 min (a), 15 min (b).

As previously mentioned, the EMW absorption capacity of a material is not only determined by *α*, but also by the combined effects of *α* and impedance matching. The calculation process for impedance matching is shown in eqn (1) and (2). As illustrated in Fig. 8(a)–(d) and S6,† the impedance matching of the composites significantly improves with increasing CNTs content in the MXene@SiO_2_–CNTs composites. Among these, the composite with a 15 minutes reaction time demonstrates the best impedance matching. This improvement is attributed to the 3D structures formed by SiO_2_ and CNTs on the MXene. First, the presence of SiO_2_ and the growth of CNTs introduce multi-scale interfaces, enhancing Maxwell–Wagner polarization. Additionally, the conductivity of CNTs contributes to high conductive loss, while their dispersion within the hybrid material increases interface resistance, which in turn induces dielectric loss. The semi-conductive properties of SiO_2_ further optimize impedance matching. As a result, the hybrids exhibit significantly improved EMW absorption capacity.

**Fig. 9:**
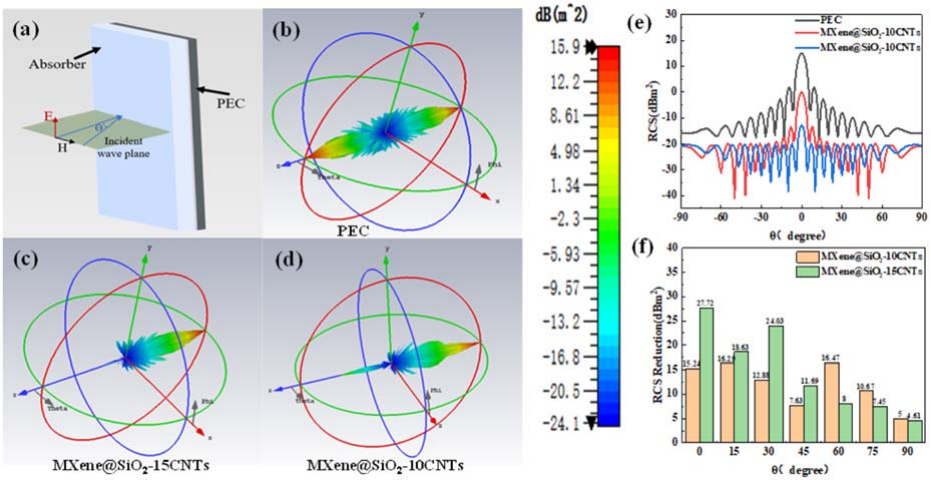
3D RCS models (a–d), 2D RCS (e) and RCS reduction (f) of samples.

In order to investigate the potential application of composite MXene@SiO_2_–CNTs hybrids in practical scenarios, radar scattering cross section simulation calculations are performed using CST Studio. As shown in Fig. 9(a), the model used consists of a perfect conductivity backsheet (PEC, 1.0 mm) and MXene@SiO_2_–CNTs hybrid material as a coating (2.0 mm) with dimensions of 180 mm × 180 mm. Radar scattering cross section (RCS) is an important indicator of stealth performance, the RCS value of a target is calculated by capturing the echo strength. When the RCS value is small, the target will be lost by the radar, which means the stealth effect is realized.

As shown in Fig. 9(b)–(d), the PEC plate has the strongest scattered signal because the pure metal plate reflects almost all the incident waves. MXene@SiO_2_–15CNTs has the smallest scattering signal. Fig. 9(e) and (f) show the 2D distribution of the RCS values and the RCS reduction values. MXene@SiO_2_–15CNTs has RCS values below −15 dB over the angular range of −90° to 90°. The RCS value of MXene@SiO_2_–15CNTs is the lowest at −12.63 dBm^2^ when the electromagnetic wave is incident perpendicularly to the absorber. The RCS reduction is 27.72 dBm^2^, which is in agreement with the excellent wave absorbing properties discussed earlier. This proves its excellent radar electromagnetic wave attenuation capability and its great potential in practical applications.

### EMW absorption mechanism of the MXene@SiO_2_–CNTs hybrids

**Fig. 10:**
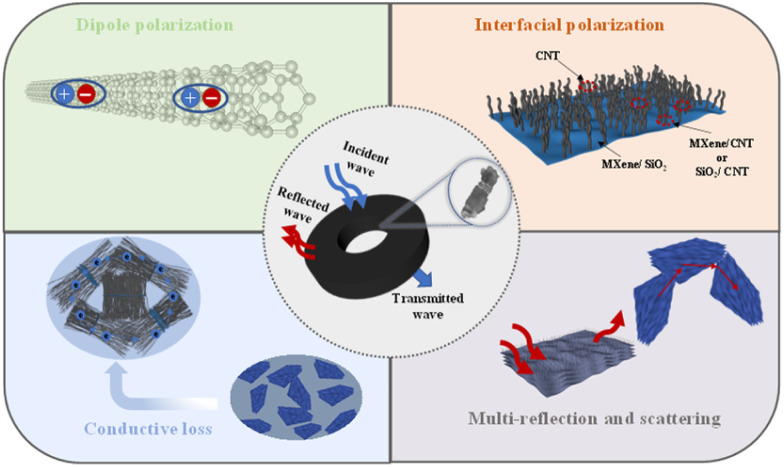
Schematic of EMW absorption mechanisms for the MXene@SiO_2_–CNTs composites.

Based on the analysis, the EMW absorption mechanism of MXene@SiO_2_–CNTs composites can be summarized in Fig. 10. Initially, CNTs with their large specific surface area, are evenly distributed on the surface of the accordion-like MXene coated with SiO_2_, forming a robust 3D conductive network. The double-layer coating of CNTs and SiO_2_ ensures good impedance matching, allowing EMW to penetrate the absorber rather than reflect off its surface. Furthermore, the CNTs prevent the stacking of MXene lamellae and serve as bridges between the layers, creating additional conductive pathways that effectively dissipate the energy of trapped EMW, thereby significantly enhancing the composites' conductive loss. Additionally, the 3D structure introduces numerous functional groups and defects, which act as multiscale interfaces to induce Maxwell–Wagner polarization and enhance dielectric loss. As a result, the MXene@SiO_2_–CNTs composites exhibit substantially improved EMW absorption performance compared to previously reported MXene–CNTs composites, as detailed in Table 1.

**Table 1:** Microwave absorption performance of various MXene–CNTs-related hybrids or structures reported in the literature

Material	Thickness (mm)	RL_min_ (dB)	Bandwidth (GHz) (RL < −10 dB)	Ref.
Ti_3_C_2_T_*x*_MXene/CNTs/Fe_3_O_4_	2.0	−40.1	5.80	55
MXene–CNTs/Ni	2.4	−56.4	—	49
MXene/CNTs microspheres	2.7	−45.0	—	20
C/TiO_2_/α-Fe	3.5	−45.1	—	56
MXene/Ni/N–CNTs	1.49	−57.8	2.08	57
Co/CNTs–MXene@CF	2.52	−61.5	5.08	47
MXene/CoNi/N–CNTs	3.8	−52.6	0.72	28
TiO_2_/C/Co@C(CNTs)-4	1.8	−41.4	5.06	58
Ti_3_C_2_T_*x*_/CNTs/TiO_2_	1.4	−44.6	4.20	59
Ti_3_C_2_@CNTs	3.5	−48.1	2.30	60
MXene@SiO_2_–15CNTs	2.1	−48.38	5.47	This work

## Conclusions

In summary, MXene@SiO_2_–CNTs hybrids with three-dimensional structures were successfully prepared by combining accordion-shaped MXene nanosheets with CNTs by CVD. The absorption mechanism of this nanocomposite relies on enhanced dielectric and conductive loss, achieved through its novel 3D structure. Coating MXene with SiO_2_ facilitates the uniform growth of CNTs and prevents the stacking of MXene lamellae, enabling low filler content in the absorber. By adjusting the growth density and length of CNTs during the CVD process, the dielectric properties of MXene@SiO_2_–CNTs composites can be tuned to achieve impedance matching. Experimental results demonstrate that with a filler loading of 15% and the thickness of 2.1 mm, the MXene@SiO_2_–15CNTs composite exhibits the EAB of 5.47 GHz (from 12.53 GHz to 18 GHz) and an RL_min_ of −48.38 dB. MXene@SiO_2_–CNTs composites demonstrate exceptional potential for lightweight broadband electromagnetic wave (EMW) absorption and stealth applications, owing to its facile synthesis, high absorption efficiency, low density, and good chemical stability.

## Author contributions

Zhichao Mu involved in data curation, investigation, validation, formal analysis, and writing—original draft. Lanzhi Wang involved in visualization, formal analysis, and writing—review and editing. Benhui Fan involved in methodology, validation, and writing—review and editing. Zuojuan Du involved in investigation, and resources. Jianling Yue involved in funding acquisition, and supervision. Yu Liu involved in conceptualization, funding acquisition, project administration, resources, supervision, and writing—review and editing. Xiaozhong Huang involved in supervision, resources, and methodology.

## Conflicts of interest

There are no conflicts to declare.

## Supplementary Material

RA-015-D5RA03991F-s001

## Data Availability

The data supporting this article have been included as part of the ESI.†
