# Supplementary material for: In situ growth of carbon nanotubes on MXenes for high-performance electromagnetic wave absorption
Source: RSC Adv. 2025 Jul 24;15(32):26506–14. doi: 10.1039/d5ra03991f (PMC12287912; doi:10.1039/d5ra03991f)
Supplement: RA-015-D5RA03991F-s001 [file RA-015-D5RA03991F-s001.pdf]

Supplementary Information (SI) for RSC Advances.
This journal is © The Royal Society of Chemistry 2025

PAPER

In-situ growing carbon nanotubes on MXene for high-performance electromagnetic wave absorption

Zhichao Mu,[ab] Lanzhi Wang,[ac] Benhui Fan, [d] Zuojuan Du,[ab] Jianling Yue,[ab] Yu Liu,[*ab] and Xiaozhong Huang,[ab]

Two-dimensional transition metal carbides and nitrides (MXene), especially titanium carbide, is one of the ideal materials for high-performance microwave absorbers. Nonetheless, its characteristics of being prone to stacking and agglomeration seriously affect its application. Moreover, its elevated electrical conductivity results in the reflection of electromagnetic waves (EMW) rather than their absorption. This study proposes a simple strategy to grow CNTs on the surface of MXene by chemical vapor deposition (CVD) technology, and introduces a SiO_2 intermediate layer to uniformly distribute CNTs on the substrate surface. Controlling the growth of CNTs by adjusting reaction time to regulate the microstructure and electromagnetic parameters of the composite materials, the wave absorption performance under low filling amount was significantly improved. The MXene@SiO_2-CNTs exhibit a minimum reflection loss of -48.38 dB at a thickness of 2.1 mm, with an effective absorption bandwidth extending to 5.47 GHz (from 12.53 GHz to 18 GHz). The radar cross-section values of MXene@SiO_2-15CNTs are all below -15 dBm², hence significantly diminishing the likelihood of radar detection of the target.

Supplementary Information

Table S1 The elemental composition of MXene@SiO_2 composites in Fig. 2 from EDS.

Phase	Atom Fraction/%			
	Ti	Si	O	C
MXene@SiO_2	13.5	0.5	25.2	60.7
MXene	11.0	0.1	18.7	70.2

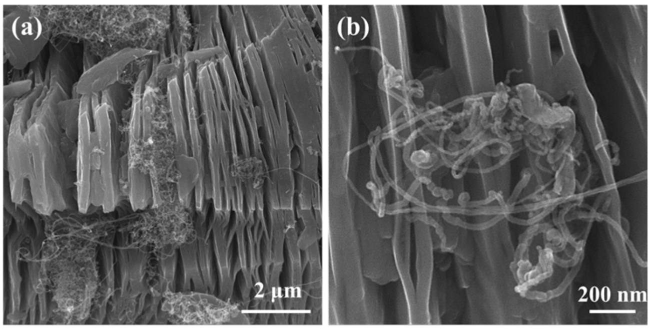

Fig. S1 SEM images of Pure MXene-15CNTs.

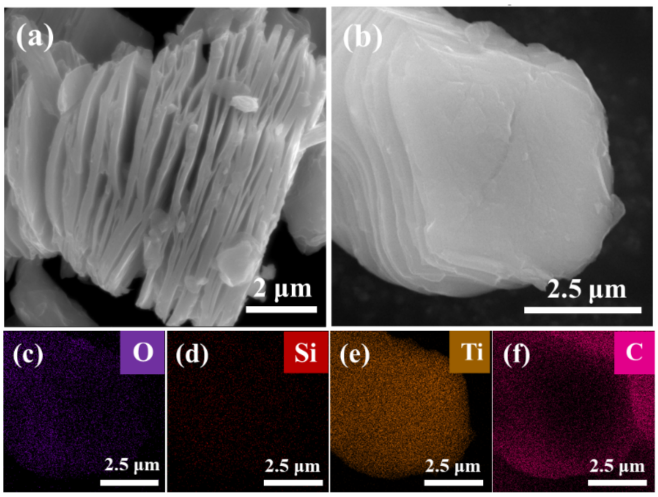

Fig. S2 SEM images of Pure MXene (a-b), EDS of images of Si, O, C, Ti elements for Pure MXene : (c-f).

[a.] Address here. State Key Laboratory of Powder Metallurgy, Powder Metallurgy Research Institute, Central South University, Changsha 410083, PR China.
[b.] Hunan Key Laboratory of Advanced Fibers and Composites, Central South University, Changsha, Hunan 410083, PR China.
[c.] Beijing Institute of Aerospace Launch Technology, Beijing 100076, PR China.
[d.] ENDSUM Research Team, Centre d'Etudes et d'Expertise sur les Risques l'Environnement, l'Aménagement et la Mobilité (Cerema), 10 chemin de la Poudrière, Le Grand-Quevilly 76120, France.

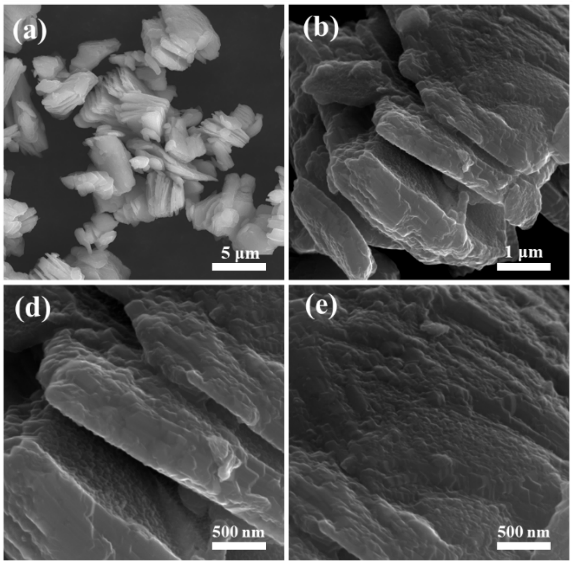

Fig. S3 SEM images of H-MXene.

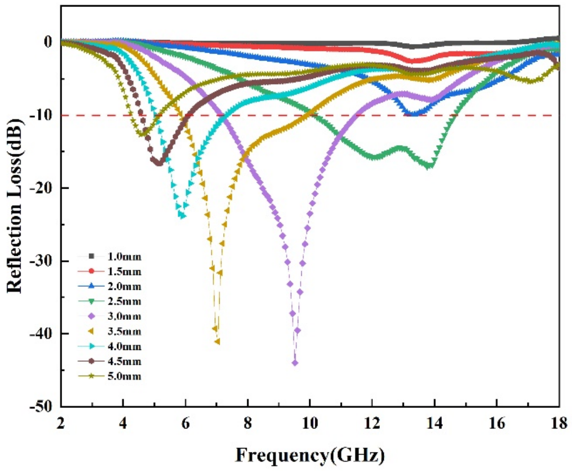

Fig. S4 Reflection loss plots of MXene@SiO_2-15CNTs with a filler concentration of 20%

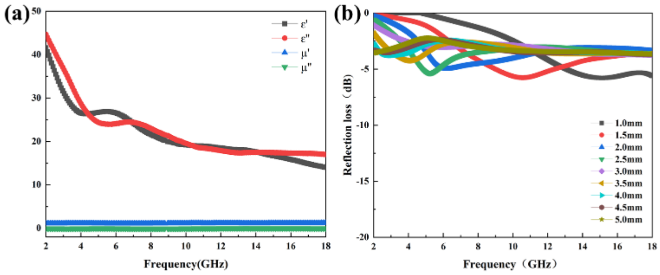

Fig. S5 the dielectric constant and reflection loss of Mxene mechanically mixed with 50%CNTs（filler concentration of 15%）

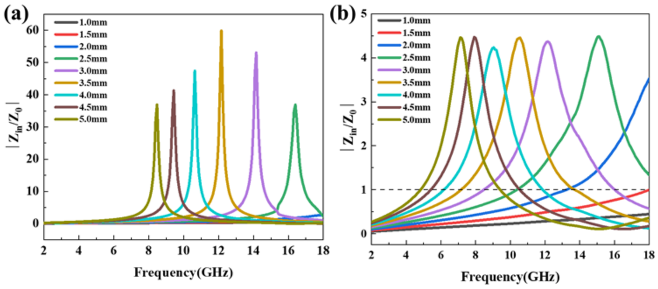

Fig. S6 Normalized characteristic impedance (Z_{in}/Z_0) plots of pure MXene (a) and MXene@SiO_2-CNTs with growth time of respectively 5min.
